# Minutes of the Biological and Microscopical Section of the Academy of Natural Sciences, Nov. 1st, 1880

**Published:** 1880-12

**Authors:** 


### MINUTES OF THE BIOLOGICAL AND MICROSCOPICAL SECTION OF THE ACADEMY OF NATURAL SCIENCES, NOV. 1st, 1880.

---

Dr. J. Gibbons Hunt, in referring to a communication made some time ago, said the announcement then made by him, that ciliated epithelia covered the intestines of vertebrated animals. was received with considerable doubt by some of the members of the section.

Since then a German journal contained a similar statement, which also verified his observations.

Dr. Hunt presented for examination some hairs taken from the head of an aged lady, which were of a beautiful carmine color. They were the short hairs underneath the gray, and covered the entire head. They presented no pathological condition. The color is permanent and of a uniform carmine tint. Under the microscope they presented no abnormal appearance.

Dr. Hunt, chairman of the committee appointed to prepare a report upon the exhibits and improvements in microscopical science, at the Fifteenth Annual Exhibition of the Section, held October 15th, 1880, read a paper, of which the following is an extract:

The hall of the Academy was comfortably filled, with perhaps, two thousand visitors, who were distributed all over the building, the museum being opened and lighted, for the fuller entertainment of our guests.

The Entomological Section contributed to our success by generously displaying a portion of their magnificient collection of butterflies, moths and beetles.

One hundred and twenty-seven miscroscopes were in position. Of this number, Zentmayer, made 48; Beck, 30; Crouch, 23; Bausch and Lomb, 7; Queen & Co., 5; Hartmack, 2; Lidle & Poalk, 2; Gundlack, 1; Ross, 1; J. C. Green, 1; J. H. Stewart, 1; unknown, 1.

Your committee can look back to the beginning of microscopy in Philadelphia, and, at that time, and for a long period after, Nachet's microscopes were the only ones—with few exceptions—then in use. Now, those inferior French instruments are not reported at our meetings.

Many preparations of animal tissues, human and comparative, were shown, such as infected lung, kidney, brain, skin, etc., and some were stained with carmine, then bathed in acid and mounted in balsam, secundum artem obsoletam; and all such looked about as well as could be expected. But experience, however, is becoming convinced that it is no longer good work to stain only the bioplasts, and to fill the vessels with gelatine colored with carmine, and mount in balsam. Around every bioplast so treated is, the cell-body, larger or smaller, which remains invisible. Moreover, growing and finest vessels cannot be structurally demonstrated in that way, neither can the connecting tissues which bind all other parts together be demonstrated or studied by these old processes.

Such work has no longer any significance in the entire and correct interpretation of the tissues, but it does create abnormal stomata, and apochryphal perivascular spaces to be found only in the books. Some recent sections, double stained by Cole & Son, of London, were shown at the meeting, and they revealed more tissue elements than any other balsam mounts yet seen this side of the Atlantic.

The division of cartilage—during change towards bone—the so-called obsteoblasts, medullary cells and fat cells, all with bioplasts showing division and vacuolation; not nebulously and doubtfully, but sharply differentiated under 500°, with the binocular, could been seen, educating us more fully into a better undestanding of such things.

A beautiful slide of the young bot fly (æstrus gasterophilus) attached to a hair from the horse, is worthy of report.

Living polyps (pectinatella magnifica) and the new rotifer (apœcia emiliæ), intermediate between the lascinularia and melicerta, are reported as well chosen illustrations of uncommon pond-life.

In exhibits of plant tissues, by double staining, we saw nothing new.

A new species (cribraria dictydioides) from our park is reported on exhibition.

Good slides of microsporon furfur, probably the cause of tinea, versicolor of the skin; and of achorion schonleinii, an alleged cause of tinea favosa, or ring-worm, both difficult to obtain and preserve, are reported.

We note a marked advance in the preservation of delicate fungi, and of fresh water algæ. All such were preserved in camphor water one ounce and glycerine about six drops.

Wickerheimer's fluid is unfit for such work. Put almarine algæ in Goadby's solution, and many will be prel served unchanged.

Many beautiful polarizing objects were well shown. Æsculine and napthaline were among the most beautiful, but it is an error to exhibit these thin crystals under the binocular with the second eye-pieces, because such displays give bad definition. Exertness in microscopic display is best learned—and perhaps learned only—by much practice in the resolution of difficult test objects, because in such work greatest attention to illumination and adjustment of lenses is essential, and when that experience is once thus acquired it influences for best results all other displays We observed many binocular microscopes badly illuminated, without achromatic condensers or an equivalent. This is an error. It is not possible to get best results with the binocular without a condenser below the stage. In all microscopic work command superabundant light. In microscopes some improvements and novelties may be reported for the past year. A small folding microscope, standing very firmly when in use, costing fifteen dollars, exhibited by Queen & Co., is worthy of report.

Beck has removed the flat foot, of whatever shape, from all his instruments, and they all stand on three toes, and are, therefore, steady. His new "International" stand presents some improvements of interest. The stage is six and one-half inches in diameter, and has three-quarters of an inch lateral motion, and nearly that in vertical direction. Motion is given by a screw and rack opposed by springs, which are claimed to obviate loss of motion, a great improvement over his former stages. The stage rotates around the optical axis, and also around its own axis, by means of milled-head pinion, enabling it to be completely inverted, and a graduated circle registers the degrees as it goes round. All the illuminating apparatus, if desired, swings around a centre lever with the stage. The obliquity of illumination is registered on a large graduated circle, which is movable up and down, carrying with it all illuminating apparatus. To fix the body in any position a lever-handled clamp is attached to one trunnion. The fine adjustment is retained on the end of the body. If this microscope can be claimed as an instrument of supreme precision, it is wrong to place the fine adjustment on the body. Every time it moved the magnifying power of the instrument is changed, and an error is introduced, and the microscope is just that much not an instrument of precision. Means have been found to remedy that defect, and all possible sources of error should be eliminated from the microscope of the future, just as soon as means are found to do it.

Another large instrument made by Beck, having a stage motion of four inches in either direction, is reported.

Zentmayer's improved "Centennial" stand was shown. Its new mechanical stage, five inches in diameter, is claimed as the thinnest yet made. Light 70° obliquis is admitted beneath it. Milled heads move it vertically one and one-eighth inches, and literally one and one-quarter inches. It may be inverted, if desired, retaining still the object in the centre of rotation of the sub-stage. The plan of swinging the sub-stage is well known, and not capable of improvement. It is the most simple plan yet devised for that purpose. The fine adjustment moves the entire body without changing its length. The finish and workmanship, and the motions of the instrument, place it, in precision, simplicity and excellence, far ahead of all microscopes at our meeting. Since this microscope appeared at our Centennial exhibition for the first time, many skillful makers have aimed to secure some of its advantages by ingenious mechanical devices, but none yet have approached its classical simplicity and fewness of parts. This instrument marked a new departure in the construction of microscopes, and without injustice your committee can say no less.

We report two lamps attached to microscopes in place of the mirrors. One was an ordinary Beck's lamp, fitted with a carrier for the purpose. The other, made by Zentmayer, was three inches high, including chimney of blue glass, and about one and one-quarter inch wide. It could be adjusted without rack in every direction, gives out no perceptible heat of a hot night, and will burn without refilling for six years. With Wenham's modified binocular prism it gives superabundant light in both fields with a twentieth objective. With his ordinary prism, it fills equally well both fields with all lenses up to a fifth. It requires only a slight addition to the ordinary condenser to make it very desirable. It is elegantly made.—*Medical and Surgical Reporter.*

